# Antimicrobial and Antibiofilm Activity of Marine *Streptomyces* sp. NBUD24-Derived Anthraquinones Against MRSA

**DOI:** 10.3390/md23080298

**Published:** 2025-07-25

**Authors:** Yuxin Yang, Zhiyan Zhou, Guobao Huang, Shuhua Yang, Ruoyu Mao, Lijian Ding, Xiao Wang

**Affiliations:** 1 Health Science Center, Ningbo University, Ningbo 315211, China; 15757626225@163.com (Y.Y.); 236002962@nbu.edu.cn (Z.Z.); hgb373792959@163.com (G.H.); 15958986181@163.com (S.Y.); 2 Gene Engineering Laboratory, Feed Research Institute, Chinese Academy of Agricultural Sciences, Beijing 100081, China

**Keywords:** marine natural products, *Streptomyces tauricus* NBUD24, MRSA, antibacterial, antibiofilm

## Abstract

Antimicrobial resistance (AMR) has emerged as a global health crisis, with methicillin-resistant *Staphylococcus aureus* (MRSA) representing one of the most clinically significant multidrug-resistant pathogens. In this study, three structurally unique anthracycline derivatives—keto-ester (**1**), 4-deoxy-ε-pyrromycinone (**2**), and misamycin (**3**)—were first isolated and characterized from the fermentation broth of the marine-derived *Streptomyces tauricus* NBUD24. These compounds exhibited notable antibacterial efficacy against MRSA, with minimum inhibitory concentrations (MICs) ranging from 16 to 32 µg/mL. Cytotoxicity assays confirmed their safety profile at therapeutic concentrations. The biofilm formation assay demonstrated that 4-deoxy-ε-pyrromycinone inhibited biofilm formation of MRSA ATCC43300, with an inhibition rate of 64.4%. Investigations of antibacterial mechanisms revealed that these compounds exert antibacterial effects primarily through disruption of bacterial cell wall integrity and destruction of DNA structure. These findings underscore the potential of marine-derived microbial metabolites as promising scaffolds for developing next-generation antimicrobial candidates to combat drug-resistant infections.

## 1. Introduction

Methicillin-resistant *Staphylococcus aureus* (MRSA), the primary pathogen causing soft tissue and skin infections, bacteremia, and joint infections [1], is listed as one of the six multidrug-resistant pathogens requiring urgent attention by the WHO [2]. The Antibiotic Resistance Threats Report released by the US Centers for Disease Control and Prevention (CDC) shows that the number of cases of serious invasive diseases and deaths caused by MRSA is estimated to reach 72,000 and 10,000, respectively, in the US each year [3,4]. The treatment of methicillin-resistant *Staphylococcus aureus* (MRSA) poses significant challenges due to the development of antimicrobial tolerance and persistence among clinically used agents [5]. Vancomycin and linezolid, regarded as last-line therapeutics for MRSA infections [6], have seen their efficacy compromised by the emergence of corresponding resistant strains following increased clinical utilization [7]. Therefore, there is an urgent need to develop new drugs with novel antibacterial mechanisms to overcome antibiotic resistance problems.

The vast ocean covers nearly 70% of the Earth’s surface [8]. Its extreme conditions, such as high salinity, high pressure, and low oxygen [9,10], have driven marine organisms to evolve diverse secondary metabolites with remarkable bioactivities, demonstrating significant potential for antibiotic development [11]. In our previous studies, three compounds, Quinosumycin [12], Xiamycin, and Chloroxiamycin [13], were isolated from *Streptomyces diastaticus* NBU2966 and *Streptomyces* sp. NBU3429 and characterized. These compounds demonstrated potent antibacterial activity against MRSA, with minimum inhibitory concentrations (MICs) ranging from 8 to 32 µg/mL. Quinosumycin represents the first reported heterodimeric scaffold featuring a thioether-bridged quinolinone–quinazolinone structure, demonstrating selective anti-MRSA activity [12]. Two indole sesquiterpene compounds, Xiamycin and its chlorinated metabolite, Chloroxiamycin, which were first isolated from the fermentation broth of marine *Streptomyces* sp. NBU3429, exhibited antibacterial/antibiofilm activity against MRSA [13]. These reports indicate that marine *Streptomyces* are an important source of potential antibacterial compounds, offering broad prospects for developing novel therapeutics against resistant pathogens.

As part of our ongoing research on bioactive *actinomycetes*, we isolated *Streptomyces tauricus* NBUD24 from the tissue of the mesophotic zone sponge (*Dasychalina* sp.). From this strain, three anthracycline derivatives—keto-ester (**1**), 4-deoxy-ε-pyrromycinone (**2**), and misamycin (**3**)—were obtained and structurally characterized. Herein, we report the isolation, structural elucidation, and evaluation of the antibacterial and antibiofilm activities of compounds **1**–**3**, along with a preliminary investigation of their mechanism of action.

## 2. Results

### 2.1. Structures of Compounds

**Figure 1:**
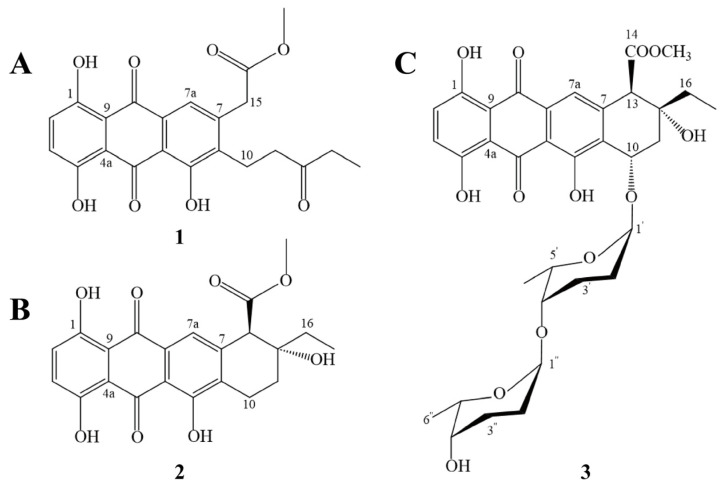
Structure of keto-ester (**A**), 4-deoxy-ε-pyrromycinone (**B**), and misamycin (**C**).

As shown in Figure 1, the structures of compounds **1**–**3** were identified as keto-ester, 4-deoxy-ε-pyrromycinone, and misamycin. The ^1^H NMR spectrum, ^13^C NMR spectrum, UV spectrum, and HRESIMS spectrum of these compounds are shown in Supplementary Figures S1–S3. In addition, the ^1^H and ^13^C NMR data of compounds **1**–**3** in CDCl_3_ are shown in Supplementary Table S1.

The molecular formula of **1** (Figure 1A) was determined as C_22_H_21_O_8_ (*m*/*z* 413.1239 [M + H]^+^) by HRESIMS (Figure S1). The ^1^H NMR spectrum (600 MHz, CDCl_3_; Table 1) of compound **1** exhibited two methyl singlets at *δ*_H_ 1.28 (H_3_-14) and 1.45 (H_3_-17), three olefinic methine protons at *δ*_H_ 6.32 (H-2), 6.15 (H-3), and 5.87 (H-7a), and four methylene groups at *δ*_H_ 2.65 (H_2_-10), 2.82 (H_2_-11), 3.12 (H_2_-13), and 2.35 (H_2_-15). Concurrently, the ^13^C NMR spectrum (150 MHz, CDCl_3_; Table 1) revealed 22 distinct carbon signals, comprising 2 methyl carbons (*δ*_C_ 8.0, 52.6), 3 olefinic methine carbons (*δ*_C_ 122.0, 129.6, 130.1), and 13 quaternary carbons, including an ester carbonyl (*δ*_C_ 170.8) and a ketone carbonyl (*δ*_C_ 186.3). Comparative analysis with published NMR data for anthracycline derivatives conclusively identified compound **1** as a keto-ester [14].

The molecular formula of **2** (Figure 1B) was determined as C_22_H_21_O_8_ (*m*/*z* 413.1239 [M + H]^+^) by HRESIMS (Figure S2). Comparative analysis with published NMR data for anthracycline analogues conclusively identified compound **2** as 4-deoxy-ε-pyrromycinone [15].

The molecular formula of **3** (Figure 1C) was determined as C_34_H_40_O_13_ (*m*/*z* 657.2586 [M + H]^+^) by HRESIMS (Figure S3). Comparative analysis with the literature NMR data for structurally related anthracyclines conclusively established compound **3** as misamycin [16].

### 2.2. MICs

The antibacterial activities of keto-ester, 4-deoxy-ε-pyrromycinone, and misamycin were tested by determining MIC values using the broth microdilution method. The MIC values of these compounds were measured against MRSA ATCC43300 and *Escherichia coli* (*E. coli*) ATCC25922, with vancomycin and polymyxin B as positive controls. As shown in Table 1, these compounds showed varying degrees of inhibition against MRSA ATCC43300 (MIC 16–32 µg/mL). However, the MIC values for *E. coli* ATCC25922 exceeded the maximum test range.

### 2.3. Cytotoxicity

**Figure 2:**
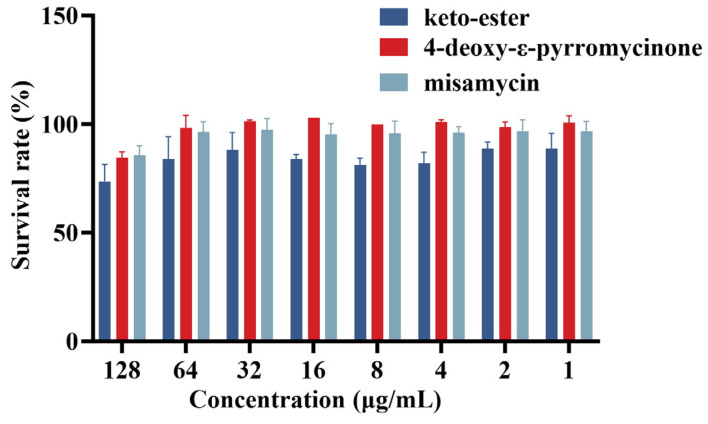
The toxicity of compounds against the RAW 264.7 cells.

The toxicity of compounds to macrophagocyte RAW 264.7 cells was determined. As illustrated in Figure 2, all compounds exhibited cytotoxicity to varying degrees at the highest test concentration. Under the treatment of keto-ester, 4-deoxy-ε-pyrromycinone, and misamycin at the concentrations of 128 µg/mL, the cell survival rates were 73.5%, 84.7%, and 85.6%, respectively. Within the test concentration range (1–128 µg/mL) of keto-ester, the cell survival rate was 73.5–88.8%. Except at the highest test concentration, the cell survival rates of 4-deoxy-ε-pyrromycinone and misamycin were both higher than 95%. 4-Deoxy-ε-pyrromycinone and misamycin can be used safely within the normal antibacterial range.

### 2.4. Scanning Electron Microscopy (SEM) Observation of Bacterial Morphology

**Figure 3:**
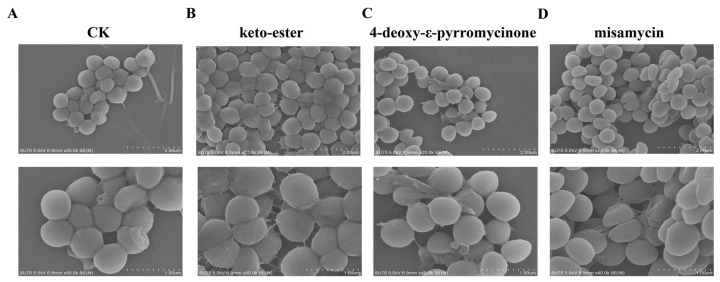
Effects of compounds on cell wall and membrane. (**A**–**D**) Scanning electron microscopy (SEM) analysis of MRSA ATCC43300 treated with 4× MIC of compounds. CK: treated with an equal volume of DMSO.

To investigate the impacts of compounds on bacterial cell walls, the cell morphology of MRSA ATCC43300 was visualized by SEM. As shown in Figure 3A–D, the MRSA ATCC43300 of the control group (CK) exhibited normal morphology and a smooth surface. After 2 h of exposure to 4× MIC compounds (keto-ester and 4-deoxy-ε-pyrromycinone: 64 µg/mL; misamycin: 128 µg/mL), shrinkage, bulging of bullae, and filamentous adherent material appeared on the bacterial surface. These results indicated that these compounds could damage the cell wall of MRSA ATCC43300 and exert antibacterial activity through wall damage.

### 2.5. Effects on Bacterial Genomic DNA

**Figure 4:**
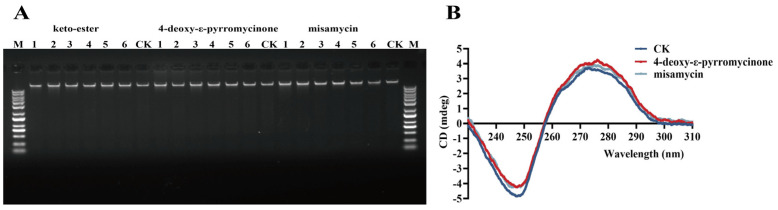
Interaction of compounds with MRSA ATCC43300 genomic DNA. (**A**) Interactions of compounds with MRSA ATCC43300 genomic DNA, which were tested by a gel migration assay. M: DNA marker; 1–6: the concentrations of compounds were 64, 32, 16, 8, 4, and 2 µg/mL, respectively; CK: treated with an equal volume of DMSO. (**B**) CD spectra of genomic DNA from MRSA ATCC43300 in the presence of keto-ester, 4-deoxy-ε-pyrromycinone, and misamycin. The concentrations of compounds and DNA were 64 and 150 µg/mL, respectively.

DNA migration was assessed by electrophoresis on a 1% agarose gel. As can be seen in Figure 4A, the bacterial genome was not subjected to a blocking effect, and thus, the compounds had a relatively minor impact on bacterial DNA-binding ability. Then we speculated whether the compounds could damage the secondary structure of DNA. Cyclization of the hexacyclic scaffold exhibited negligible effects on MIC values, and we aimed to investigate whether the presence of glycosyl moieties would interfere with the secondary structural integrity of DNA. Therefore, we used 4-deoxy-ε-pyrromycinone and misamycin for a CD spectrometer assay. As displayed in Figure 4B, the positive and negative peaks of normal bacterial DNA occurred at about 275 nm and 245 nm. After treatment with compounds, although the overall shape of the DNA chromatogram was similar to the control group, the peak value at 245 nm decreased for both drug-treated groups, indicating that the compounds had an impact on the structure of DNA.

### 2.6. Transcriptome Analysis of MRSA ATCC43300 Treated with Compounds

**Figure 5:**
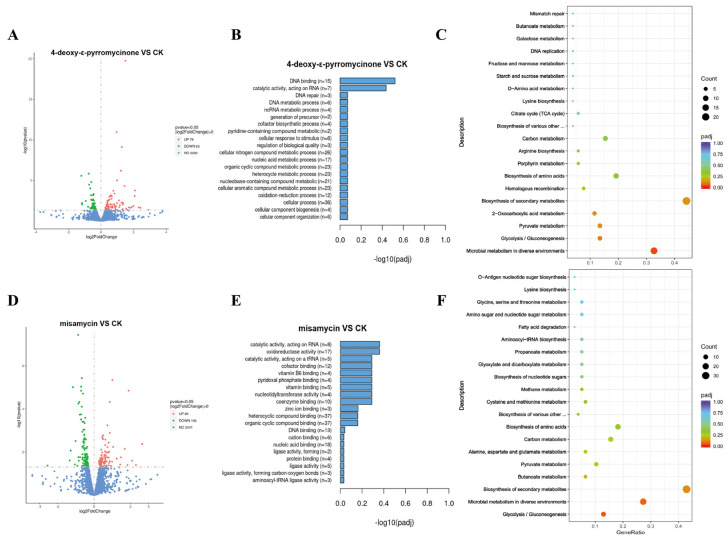
Transcriptome analysis of *S. aureus* treated with compounds (1× MIC). (**A**) Volcano plot of the significantly differentially expressed mRNA genes in *S. aureus* following treatment with 4-deoxy-ε-pyrromycinone. (**B**) GO function analysis chart of significantly differential genes in *S. aureus* following treatment with 4-deoxy-ε-pyrromycinone. (**C**) Bubble chart for the KEGG pathway enrichment of significantly upregulated and downregulated genes in *S. aureus* following treatment with 4-deoxy-ε-pyrromycinone. (**D**) Volcano plot of the significantly differentially expressed mRNA genes in *S. aureus* following treatment with misamycin. (**E**) GO function analysis chart of significantly differential genes in *S. aureus* following treatment with misamycin. (**F**) Bubble chart for the KEGG pathway enrichment of significantly upregulated and downregulated genes in *S. aureus* following treatment with misamycin.

Transcriptomic analysis provides crucial molecular insights into elucidating the antimicrobial mechanisms of many antimicrobial agents [17,18,19]. To further explore the potential antibacterial mechanisms by which the compounds act on *S. aureus*, transcriptome analysis was performed on *S. aureus* treated with compounds (1× MIC) for 4 h. As shown in Figure 5A–F, 4-deoxy-ε-pyrromycinone and misamycin treatment identified 142 (79 upregulated and 63 downregulated) and 193 (85 upregulated and 106 downregulated) significantly differentially expressed genes (DEGs, |log2(fold change)| > 1, FDR < 0.05), respectively. All of the compound treatment groups downregulated genes involved in cell wall synthesis, including *ezrA*, *mraZ*, *cwrA*, etc. [20,21,22]. It was indicated that the compounds could inhibit the formation of the bacterial cell wall, which is consistent with the results of SEM. In addition, many genes were significantly upregulated after being treated with compounds; for example, *recA*, *lexA,* and *uvrB* for DNA replication and repair systems were increased to protect cells from the corresponding stress response [23,24]. KEGG pathway analysis identified 20 metabolic pathways affected by the treatments. Notably, both compounds significantly downregulated genes associated with microbial metabolism in diverse environments and glycolysis/gluconeogenesis pathways. We speculate that the addition of the compound triggers the bacterial stress mechanism, which then regulates metabolism and DNA damage repair to adapt to the presence of the compound, ultimately ensuring the survival and proliferation of the bacteria in an unfavorable environment. These pathways are critically involved in amino acid metabolism and energy production—essential biological processes for bacterial survival. The marked suppression of these metabolic pathways suggests that the compounds likely exert their antibacterial effects by disrupting bacterial amino acid metabolism synthesis and energy homeostasis.

### 2.7. Biofilm Formation Assay

**Figure 6:**
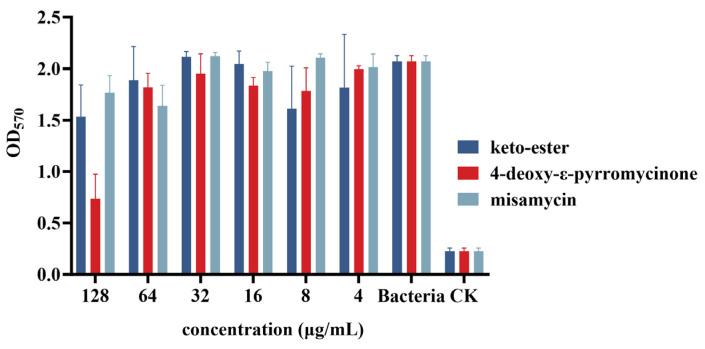
The inhibition abilities of compounds against MRSA biofilms.

As illustrated in Figure 6, at the highest tested concentration, 4-deoxy-ε-pyrromycinone demonstrated an inhibitory effect on biofilm formation with an inhibition rate of 64.4%. In comparison, the inhibition rates of the other two compounds were 25.6% and 14.4%, respectively. At lower tested concentrations (ranging from 1 to 64 µg/mL), all compounds exhibited relatively low inhibition rates against biofilm formation.

## 3. Discussion

The MICs of the compounds against MRSA ranged from 16 to 32 µg/mL, and there was no significant difference in antibacterial activity between the compounds. Based on the MIC results, all of our compounds have a parent structure—1,4,5-trihydroxy-9,10-anthraquinone. Therefore, we speculate that this structure is the key point for the compounds’ antimicrobial effect. Our result is consistent with the previous research conclusions that molecules with similar structures usually have similar drug effects [25]. Among these compounds, keto-esters show a certain structural similarity to 4-deoxy-ε-pyrromycinone, as both contain methyl and ester bond structures. Their main difference lies in whether the six-membered ring forms a closed loop, but it does not change the MIC values. The side chain of misamycin contains a unique disaccharide structure — α-narbosine B, which may increase its MIC to 32 µg/mL. None of the compounds exhibit antibacterial activity against *E. coli* ATCC25922 at a concentration of 128 µg/mL. This might be due to the presence of the outer membrane of Gram-negative bacteria, which prevents the drugs from exerting their effects.

Given the cytotoxicity associated with anthraquinones in clinical oncology practice, we conducted a comprehensive safety assessment of these compounds. The result showed that, although the compounds exhibit dose-dependent cytotoxicity, the cell survival could reach 73.3–85.6% at the highest tested concentration (128 µg/mL). When antimicrobial treatment is performed using 1× or 2× MIC concentrations, cell survival was consistently maintained above 80%. This indicates an acceptable safety profile in antimicrobial applications. In particular, 4-deoxy-ε-pyrromycinone and misamycin exhibit improved safety margins relative to keto-esters and doxorubicin, a widely used antitumor agent [26].

Subsequently, we explored the compounds’ antibacterial mechanism. The cell wall of Gram-positive bacteria is mainly a reticular scaffolding structure formed by peptidoglycan. In this study, we observe that compounds containing anthraquinone structures can disrupt the integrity of bacterial cell walls, which is consistent with the findings of Tang et al. [27]. Moreover, transcriptomic profiling demonstrated significant downregulation of *ezrA*, *mraZ*, and *cwrA* genes in compound-treated strains. The downregulation of *ezrA* and *mraZ* disrupts the FtsZ protein assembly and Z-ring formation, leading to defective cell wall synthesis during division [20,21]. Concurrently, the reduced expression of *cwrA* promotes peptidoglycan hydrolysis, interfering with the structural integrity of the cell wall [22]. Following cellular internalization, the compounds can further damage the secondary structure of genomic DNA, which extends the previous conclusions that anthraquinones are considered to be inhibitors of bacterial topoisomerases I and II [28]. Therefore, we speculate that our compounds can target the bacterial DNA topoisomerase, thereby inhibiting the replication and transcription of DNA and then blocking the expression of proteins. Hence, bacteria need to upregulate DNA-damage-repair-related genes (*recA*, *lexA*, and *uvrB*) to activate damage repair pathways and sustain vital cellular processes under stress conditions.

MRSA can form biofilms, which increase bacterial resistance to antibiotics [29]. Once these bacteria establish biofilms, it is tough to eliminate them, which is one of the main reasons for persistent and chronic infections [30]. There is an urgent clinical need to develop novel therapeutic agents effective against MRSA and its biofilm-associated infections. In our study, we find that 4-deoxy-ε-pyrromycinone shows a better antibiofilm activity. Compared with keto-esters and misamycin, 4-deoxy-ε-pyrromycinone effectively suppressed biofilm growth, achieving an inhibition rate of 64.4% at 8× MIC (128 µg/mL). We hypothesize that 4-deoxy-ε-pyrromycinone inhibits biofilm formation by decreasing mRNA levels of cwrA and *ezrA*. The downregulation of *cwrA* induced the activation of the *icaR* operon, thereby decreasing PIA production and reducing biofilm adhesion capacity [31]. The transcription inhibition of *ezrA* leads to reduced extracellular polysaccharide levels and architectural changes, resulting in impaired biofilm formation [32].

## 4. Materials and Methods

### 4.1. General Experimental Procedures

The ^13^C NMR and ^1^H chemical shifts were obtained relative to the solvent signal CDCl_3_ (*δ*_H_ 7.26/*δ*_C_ 77.16) on a Bruker AVANCE NEO 600 MHz instrument (Bruker Bio spin AG, Fällanden, Switzerland). Tetramethylsilane (TMS) was used as an internal standard. Medium-pressure liquid chromatography (MPLC) separations were carried out on a Bonna-Agela FLEXA purification system. C18 reversed-phase silica gel (50 µm, YMC Co., Ltd., Tokyo, Japan) and silica gel (200–300 mesh, Qingdao, China) were used for column chromatography. HRESIMS data were acquired using a Waters G2-XS Q-TOF mass spectrometer (Milford, MA, USA) coupled with liquid chromatography (LC/MS). Preparative HPLC was conducted on an Agilent 1260 system (Agilent Technologies, Lexington, MA, USA) equipped with a DAD and a YMC C18 column (10 × 250 mm, 5 µm, YMC).

### 4.2. Antimicrobial Agents

*Streptomyces tauricus* NBUD24 (*actinobacterial* strain) was derived from the South China Sea, China. It was obtained from tissue samples of the mesophotic zone sponge *Dasychalina* sp. The strain was defined by 16S rDNA sequence amplification (GenBank accession number PRJNA1187314) and deposited at Ningbo University, Ningbo, China.

### 4.3. Fermentation, Extraction, and Isolation

A fresh *actinomycete* colony was obtained by culturing on ISP2 plates at 25 °C for 3 days. The *strain* was subsequently inoculated in 300 mL ISP-2 medium (4.0 g/L yeast extract, 4.0 g/L glucose, 30 g/L sea salt, 10 g/L malt extract, pH 7.2). The culture flasks were incubated for 3d (28 °C, 220 rpm). The seed culture (6.0 L) was transferred to a rotary 1000 mL Erlenmeyer flask, with 300 mL of a liquid medium (9 g artificial sea salt, 1.5 g yeast extract, 3 g glucose, 1.5 g malt extract, 0.15 g CaCO_3_, 6.0 g starch). 12 days later, all fermentation broths were extracted three times with ethyl acetate (EtOAc) to generate crude extract (22.6 g).

The crude extract was subjected to silica gel column chromatography (200–300 mesh) using a stepwise gradient of ethyl acetate in petroleum ether (PE) (100:0, 95:5, 90:10, 85:15, 80:20, 70:30, 50:50, 0:100), yielding 3 major fractions (Fr. A-C). Fr. B was further fractionated on an ODS column (H_2_O/MeOH, 2:3, 0:1), yielding six subfractions (Fr.B.1-6). Subsequent purification of Fr.B.2, Fr.B.4 and Fr.B.5 by semi-preparative HPLC (34%, 53%, and 56% MeCN respectively; flow rate 2 mL/min) afforded compounds **1** (tR = 45 min, 4.8 mg), **2** (tR = 29 min, 3.5 mg) and **3** (tR = 37 min, 5.6 mg).

### 4.4. Structure Elucidation

Compound **1** was isolated as a yellow amorphous powder. Then the structure of compound **1** was identified as a keto-ester by comparing the NMR data. The ^1^H and ^13^C NMR spectroscopic data is as follows: ^1^H NMR (600 MHz, CDCl_3_) *δ* 7.75 (s, H-7a), 7.30 (d, *J* = 9.4 Hz, H-2, H-3), 3.72 (s, H-17), 3.49 (s, H-15), 3.03 (t, *J* = 7.5 Hz, H-10), 2.80 (t, *J* = 7.5 Hz, H-11), 2.44 (q, *J* = 7.3 Hz, H-13), 1.07 (t, *J* = 7.3 Hz, H-14); ^13^C NMR (150 MHz, CDCl_3_) *δ* 210.6 (C-12), 191.1 (C-5), 186.3 (C-8a), 170.8 (C-16), 161.5 (C-6), 158.4 (C-4), 157.9 (C-1), 142.5 (C-7), 137.4 (C-6a), 131.1 (C-8), 130.1 (C-2), 129.6 (C-3), 122.0 (C-7a), 114.6 (C-5a), 112.8 (C-4a), 112.7 (C-9), 52.6 (C-17), 40.5 (C-11), 39.5 (C-15), 36.0 (C-13), 21.4 (C-10), 8.0 (C-14).

### 4.5. Bacterial Strains and Cells

The strains (MRSA ATCC43300 and *E. coli* ATCC25922) were purchased from the American Type Culture Collection (Manassas, VA, USA). The RAW 264.7 cell line was donated by Dr. Guo Hua (Ningbo University).

### 4.6. MICs

MICs of the compounds were determined using the micro broth dilution method, as previously described [33]. In the assay, Mueller–Hinton (MH) broth and Luria–Bertani (LB) broth were used to culture MRSA ATCC43300 and *Escherichia coli* ATCC25922, respectively. The mid-logarithmic phase of bacteria was diluted to 1 × 10^5^ CFU/mL. The compounds were diluted with DMSO using two-fold serial dilution, with a concentration gradient from 1 to 128 µg/mL. After 2 µL of the compound and 98 µL of the bacterial suspension were added to a 96-well plate, the plate was incubated at 37 °C for 16–24 h. The MIC value is defined as the lowest concentration at which no bacterial growth is observed.

### 4.7. Cytotoxicity

The effect of compounds on the viability of murine peritoneal RAW 264.7 macrophage cells was determined by the CCK-8 method [34]. Cells were added to a 96-well plate at a density of 2.5 × 10^4^ cells/well and cultured at 37 °C for 24 h in a 5% CO_2_ air environment. Cells were exposed to serial concentrations of test compounds (1–128 µg/mL) for 24 h, with DMSO-treated cells serving as a control. Each well was supplemented with 10 µL of WST-8 solution. The plate was protected from light and cultured at 37 °C for 4 h. The absorbance at 460 nm was measured. The following formula was used to calculate cell viability: cell viability (%) = OD 460  nm of treated sample/OD 460 nm of control × 100%.

### 4.8. Scanning Electron Microscopy Observation of Bacterial Morphology

The mid-logarithmic phase of MRSA ATCC43300 (1 × 10^8^ CFU/mL) was cultured with 4× MIC compounds for 2 h at 37 °C. After three washes with PBS, the bacteria were fixed overnight at 4 °C with 2.5% glutaraldehyde. A graded series of ethanol (20–100%) was used to dehydrate bacteria, with each concentration applied for 5 min, followed by drying with CO_2_. The surface of the samples was sprayed with gold–palladium, and the bacteria were observed using an S4800 scanning electron microscope (Hitachi, Tokyo, Japan) [35].

### 4.9. Effects on Bacterial Genomic DNA

#### 4.9.1. DNA Gel Migration Assay

Genomic DNA was obtained by a bacterial genome extraction kit (Aidlab Biotechnologies Co., Ltd., Beijing, China). Compounds (2–64 µg/mL) were incubated with equal volumes of bacterial genomic DNA solution and then incubated at room temperature for 10 min. The DNA-binding effects of compounds **1**–**3** were evaluated using 0.8% agarose gel electrophoresis [36].

#### 4.9.2. Circular Dichroism Spectroscopy

Circular dichroism (CD) spectroscopy was used to further assess the DNA-binding affinity of compounds **1**–**3**. Each compound (64 µg/mL) was added to a genomic DNA solution (150 µg/mL) and then incubated at room temperature for 10 min. Measurements were conducted using a 1.0 mm path length quartz cuvette on a J-1700 CD spectrometer (JASCO, Tokyo, Japan), with spectra recorded from 220 to 320 nm (scan rate of 10 nm/min) [36].

### 4.10. Transcriptome Analysis of MRSA ATCC43300 Treated with Compounds

The mid-logarithmic phase of MRSA ATCC43300 (OD value = 0.5) was incubated with 1× MIC compounds for 4 h at 37 °C [32,33]. The bacterial suspension was centrifuged at 4 °C and 4000 rpm for 10 min, followed by two washes with PBS. The bacteria were immediately placed in liquid nitrogen and frozen for 15 min. Three biological replicates were collected for each condition. The total RNA extracted from the bacterial samples was characterized and quantified at Novogene (Beijing, China).

### 4.11. Biofilm Formation Assay

The crystalline violet method was used to study the biofilm formation inhibition of the compounds. The mid-logarithmic phase cultures of *Staphylococcus aureus* ATCC43300 were diluted to a concentration of 1 × 10^8^ CFU/mL by tryptic soy broth (TSB) medium, with test compounds at final concentrations spanning 1–128 µg/mL. The compound–bacterium mixtures were in a 96-well plate at 37 °C for 24 h [27,28]. Next, the biofilm was washed twice with PBS and fixed for 15 min with 2.5% glutaraldehyde. The biofilm was stained for 0.5 h with 0.1% crystal violet and dried overnight. After drying, the sample was dissolved in 95% ethanol for 30 min and then measured at the absorbance of 570 nm.

## 5. Conclusions

In this study, we identified three anthracycline derivatives—keto-ester (**1**), 4-deoxy-ε-pyrromycinone (**2**), and misamycin (**3**)—that exhibited antibacterial and antibiofilm activity against MRSA. Cytotoxicity assays confirmed their favorable safety profiles at therapeutically relevant concentrations. Mechanistic investigations revealed that these compounds disrupt the bacterial cell wall, induce DNA damage, and impair metabolic synthesis, collectively leading to bacterial cell death. Our findings highlight the potential of these anthracyclines as novel dual-functional agents capable of simultaneously targeting bacterial viability and biofilm formation, offering a promising way for combating MRSA infections.

## Figures and Tables

**Table 1:** The MICs of compounds against MRSA ATCC43300 and *E. coli* ATCC25922.

Drugs	MICs			
	ATCC43300		ATCC25922	
	(µg/mL)	(µM)	(µg/mL)	(µM)
Keto-ester	16	38.70	>128	>309.61
4-Deoxy-ε-pyrromycinone	16	38.70	>128	>309.61
Misamycin	32	48.73	>128	>194.92
Vancomycin	1	0.69	/	/
Polymyxin B	/	/	2	1.54

## Data Availability

The original contribution of this research is included in the article and the Supplementary Materials. Analytical data included in this study are available from the corresponding author upon reasonable request.

## Supplementary Materials

The following supporting information can be downloaded at https://www.mdpi.com/article/10.3390/md23080298/s1: Figure S1: The ^1^H NMR spectrum, ^13^C NMR spectrum, UV spectrum, and HRESIMS spectrum of keto-ester.; Figure S2: The ^1^H NMR spectrum, ^13^C NMR spectrum, UV spectrum, and HRESIMS spectrum of 4-deoxy-ε-pyrromycinone. Figure S3: The ^1^H NMR spectrum, ^13^C NMR spectrum, UV spectrum, and HRESIMS spectrum of misamycin. Table S1: The ^1^H and ^13^C NMR data of compounds **1-3** in CDCl_3_ (*δ* in ppm).
