# Supplementary material for: Antimicrobial and Antibiofilm Activity of Marine Streptomyces sp. NBUD24-Derived Anthraquinones Against MRSA
Source: Mar Drugs. 2025 Jul 25;23(8):298. doi: 10.3390/md23080298 (PMC12387178; doi:10.3390/md23080298)
Supplement: Supplementary file 1 [file marinedrugs-23-00298-s001.zip › marinedrugs-3730204-supplementary.pdf]

Supplementary materials

Antimicrobial and Antibiofilm Activity of Marine *Streptomyces sp.* NBUD24-Derived Anthraquinones against MRSA

Yuxin Yang[1], Zhiyan Zhou[1], Guobao Huang[1], Shuhua Yang[1], Ruoyu Mao[2*], Lijian Ding[1*], Xiao Wang[1*]

1 Health Science Center, Ningbo University, Ningbo, Zhejiang 315211, China.

2 Gene Engineering Laboratory, Feed Research Institute, Chinese Academy of Agricultural Sciences, Beijing 100081, China.

** Correspondences:

Professor PhD. Ruoyu Mao:maoruoyu@caas.cn;
Professor PhD. Lijian Ding:dinglijian@nbu.edu.cn;
Professor PhD. Xiao Wang:wangxiao@nbu.edu.cn.

SUPPORTING INFORMATION

Supplementary 1: Figures

Figure S1. The ^1H NMR spectrum, ^{13}C NMR spectrum, UV spectrum, and HRESIMS spectrum of keto-ester.

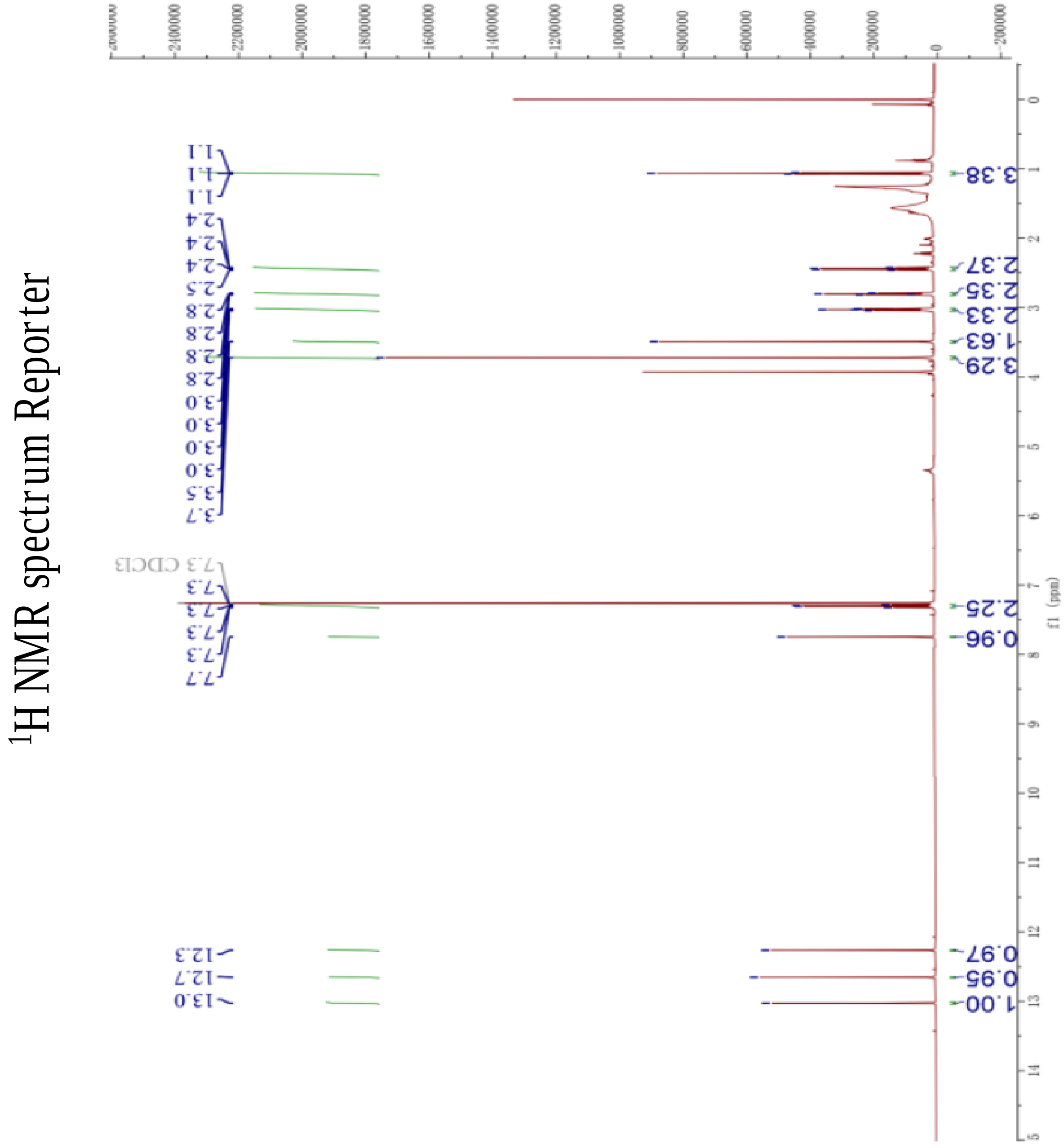

^{13}C NMR spectrum Reporter

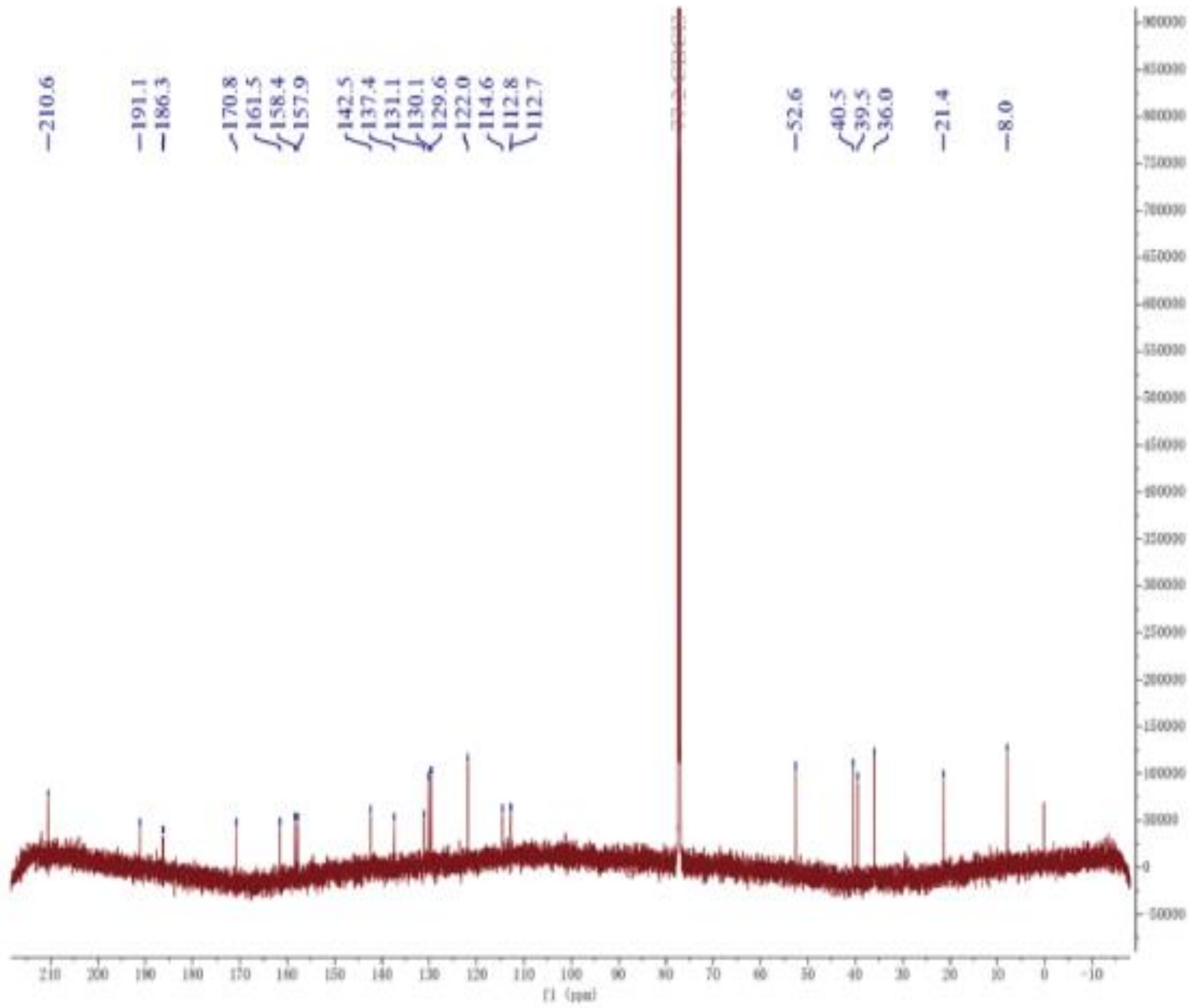

UV spectrum

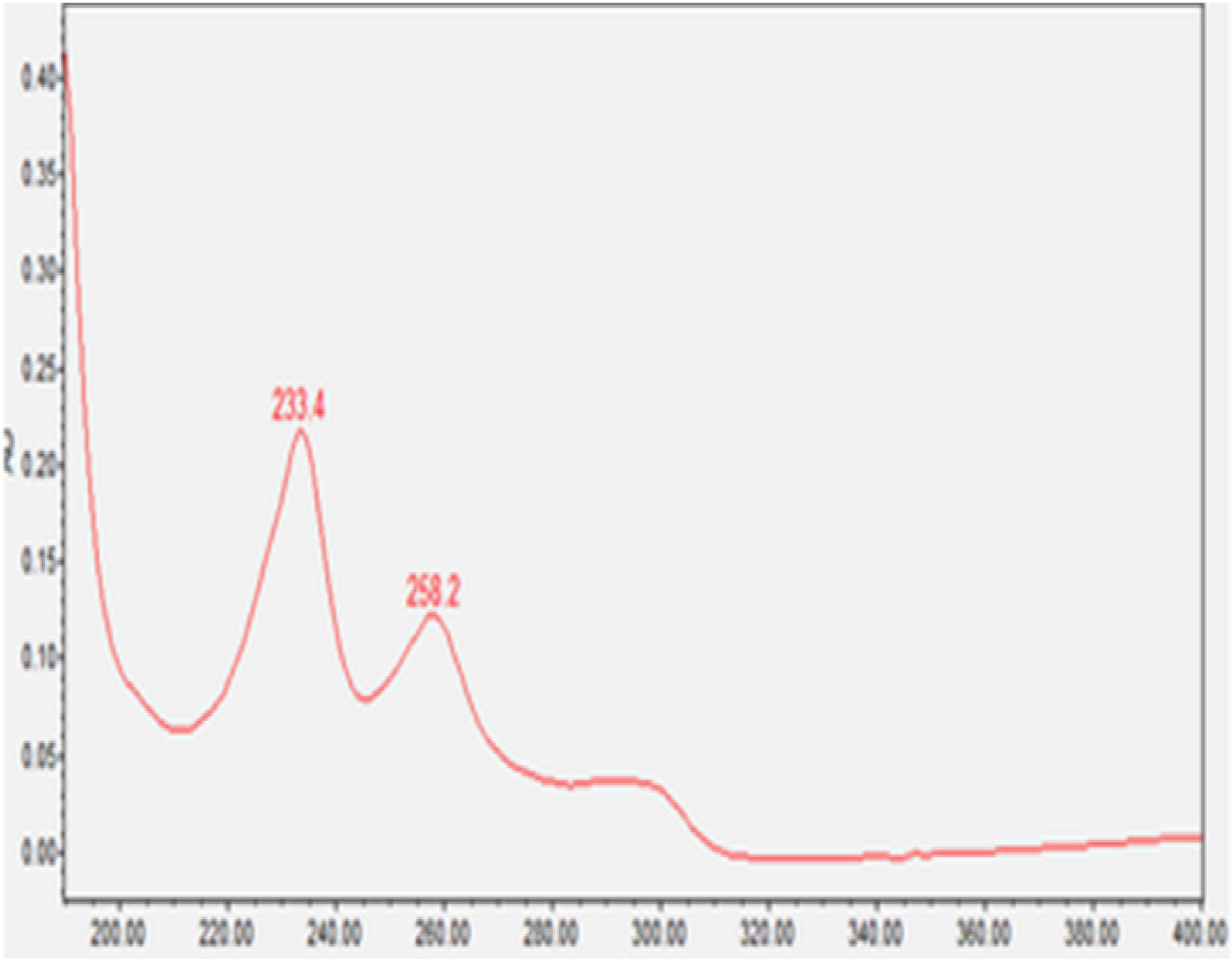

nm

HRESIMS spectrum

Single Mass Analysis
Tolerance = 5.0 mDa / DBE: min = -1.5, max = 50.0
Element prediction: Off
Monoisotopic Mass, Even Electron Ions
1 formula(e) evaluated with 1 results within limits (up to 50 closest results for each mass)
Elements Used:
C: 22-23 H: 21-21 O: 8-8 Na: 0-1

Mass	Calc. Mass	mDa	PPM	DBE	Formula	C	H	O	N...
413.1239	413.1236	0.3	0.7	12.5	C22 H21 O8	22	21	8	

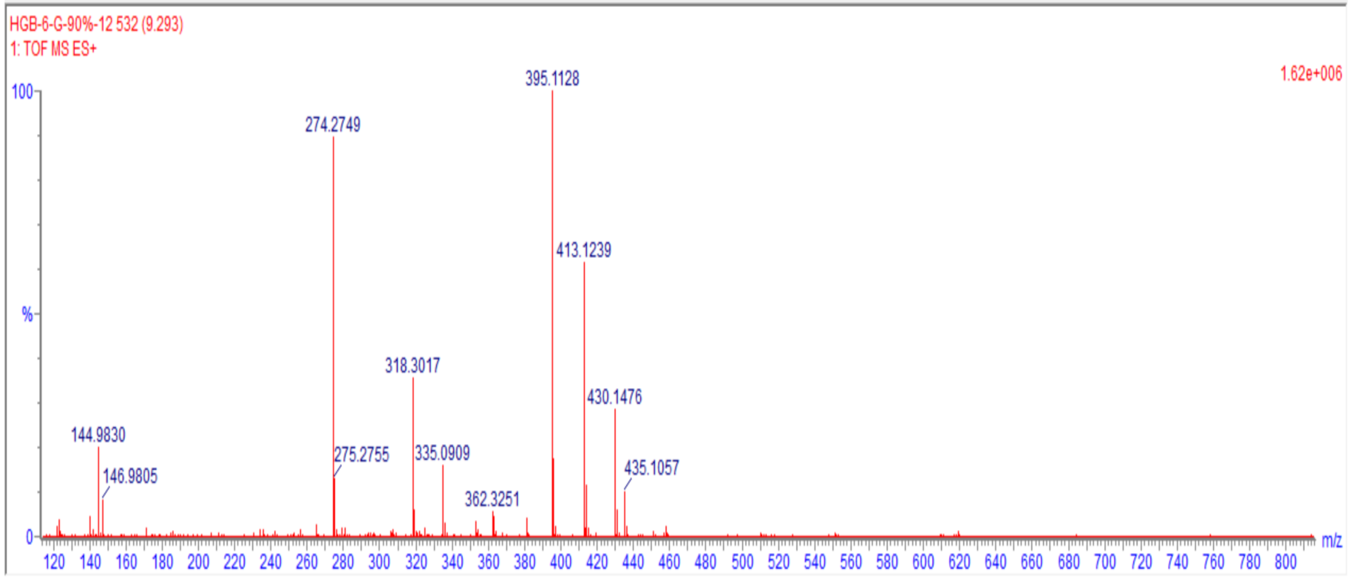

Figure S2. The ^{1}H NMR spectrum, ^{13}C NMR spectrum, UV spectrum, and HRESIMS spectrum of 4-deoxy-ε-pyrromycinone.

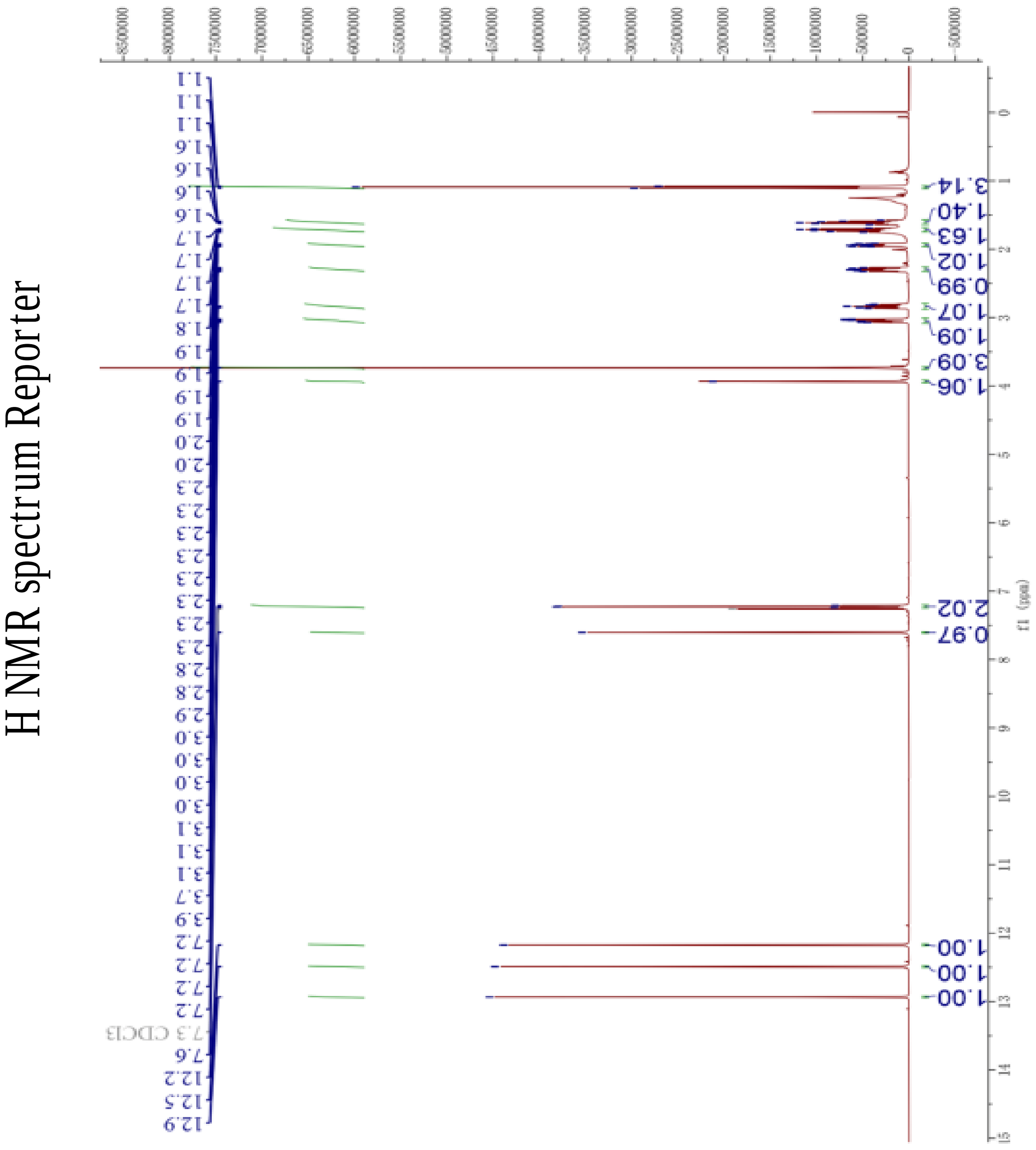

^{13}C NMR spectrum Reporter

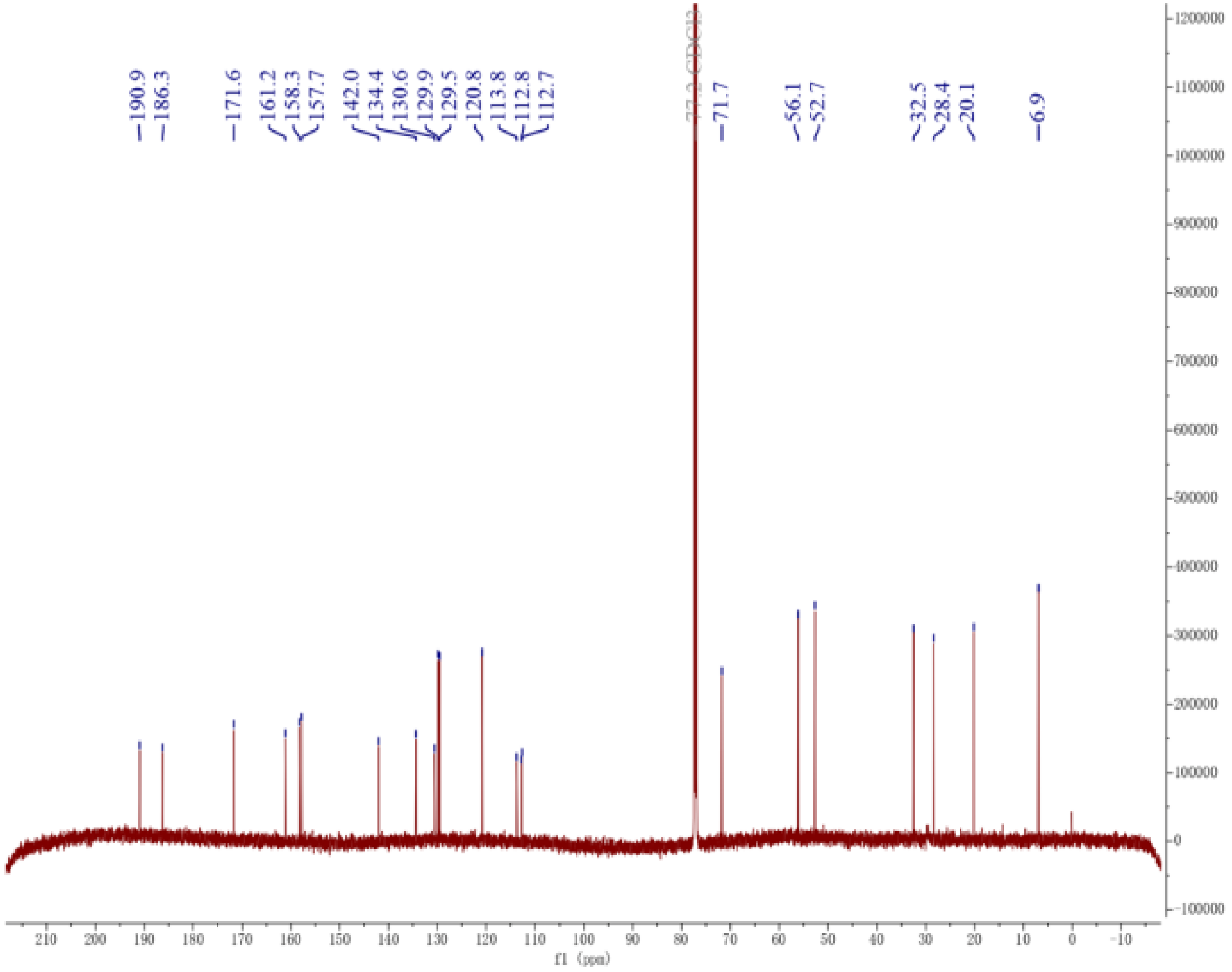

UV spectrum

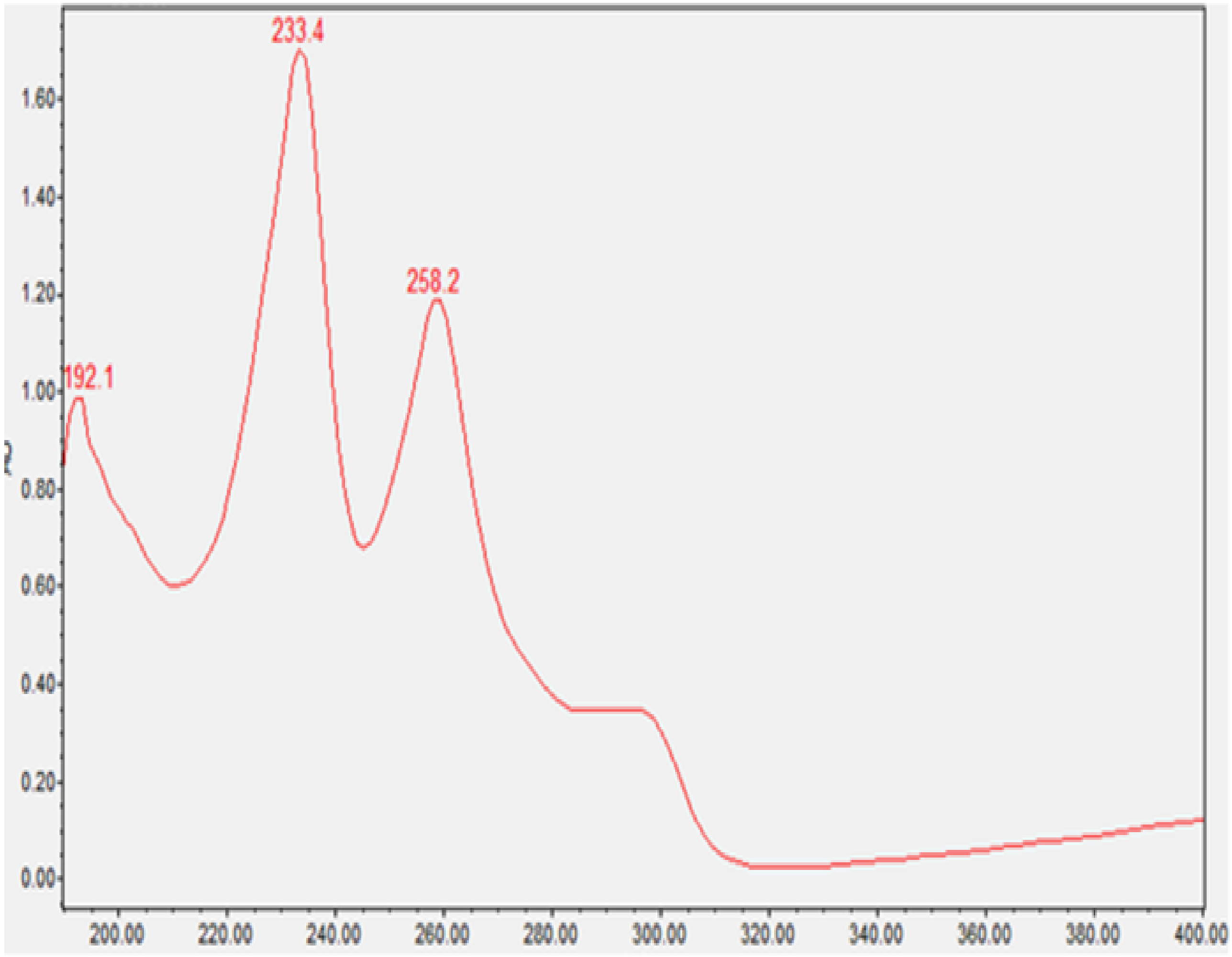

nm

HRESIMS spectrum

Single Mass Analysis
Tolerance = 5.0 mDa / DBE: min = -1.5, max = 50.0
Element prediction: Off
Monoisotopic Mass, Even Electron Ions
1 formula(e) evaluated with 1 results within limits (up to 50 closest results for each mass)
Elements Used:
C: 22-22 H: 21-21 O: 8-8 Na: 0-1

Mass	Calc. Mass	mDa	PPM	DBE	Formula	C	H	O	N...
413.1239	413.1236	0.3	0.7	12.5	C22 H21 O8	22	21	8	

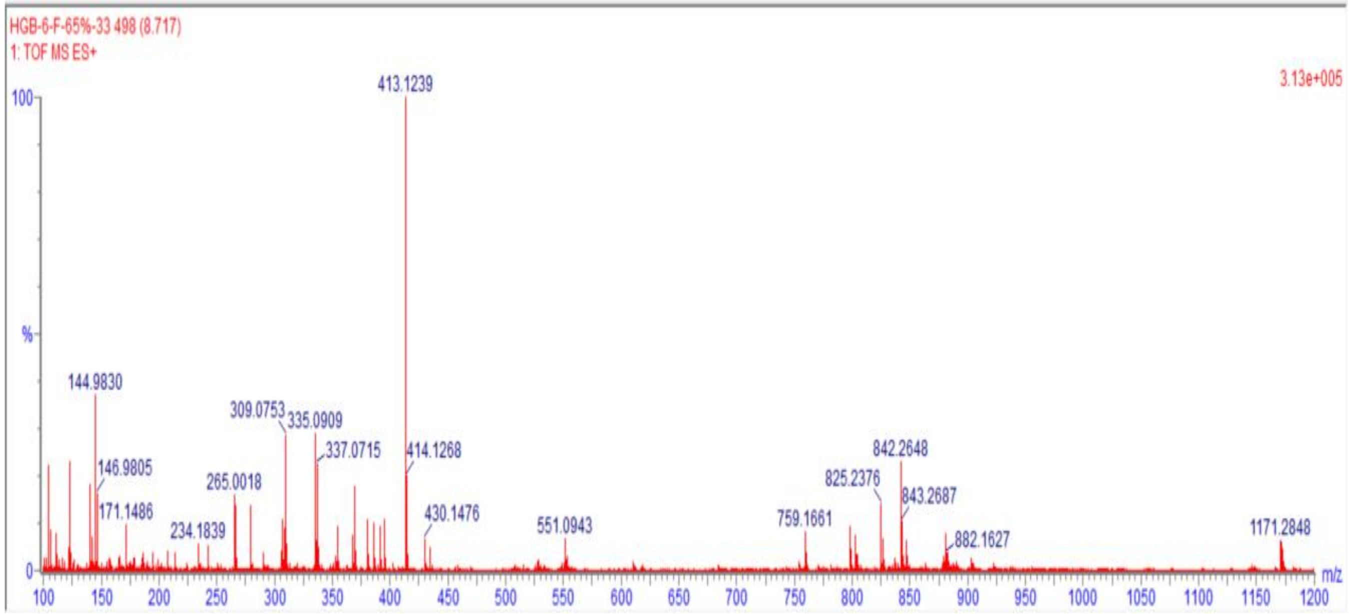

Figure S3. The ^{1}H NMR spectrum, ^{13}C NMR spectrum, UV spectrum, and HRESIMS spectrum of misamycin.

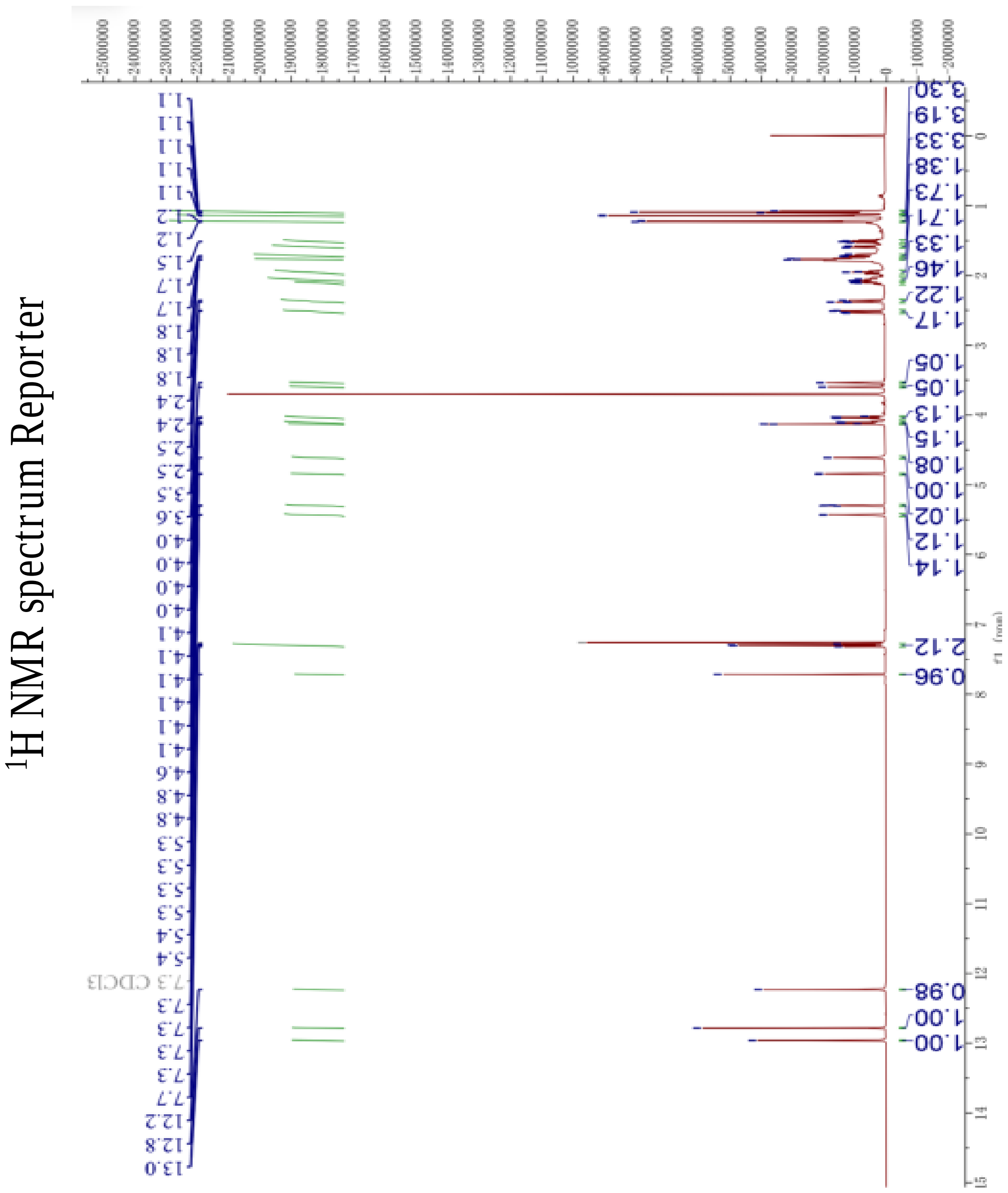

^{13}C NMR spectrum Reporter

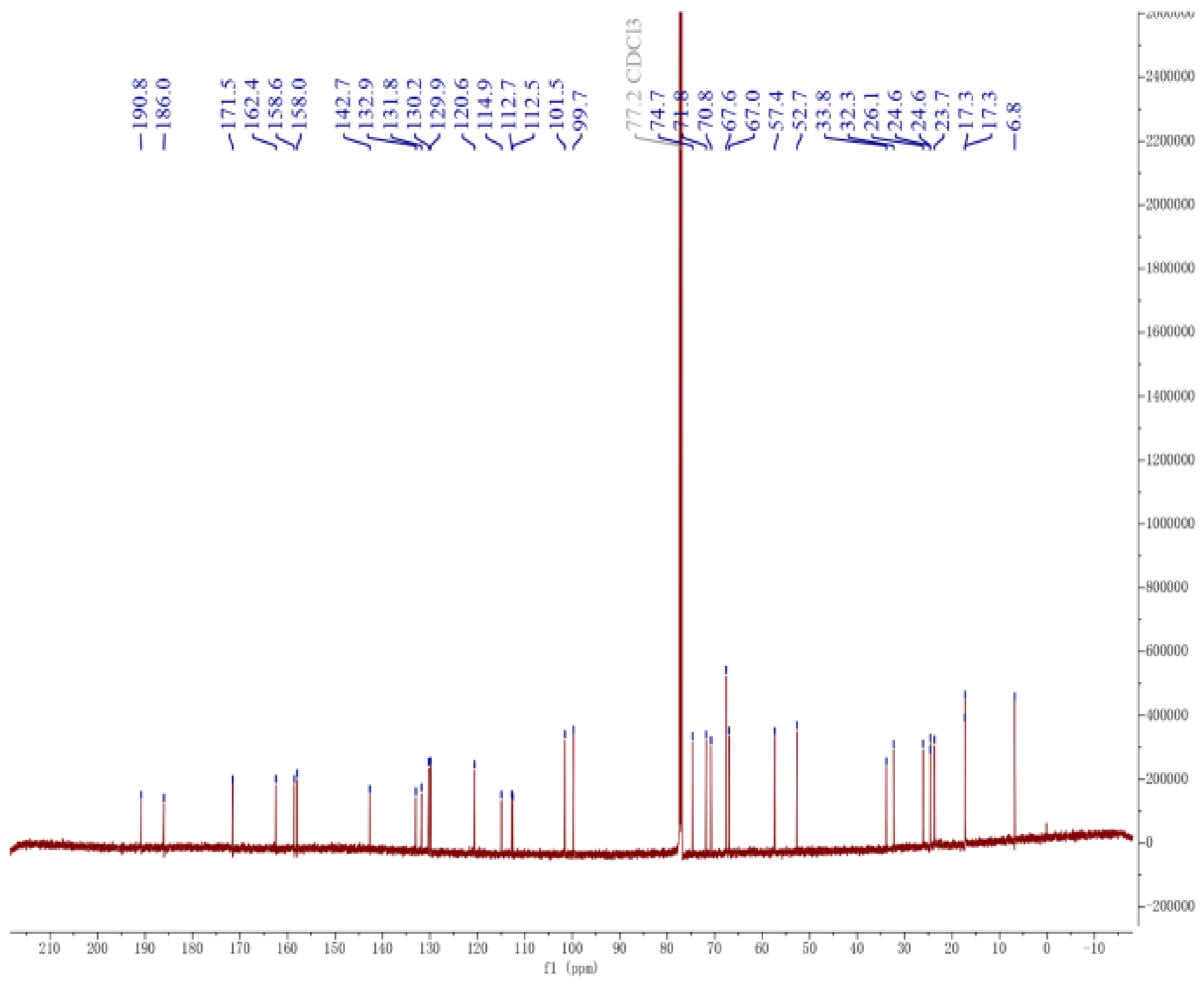

UV spectrum

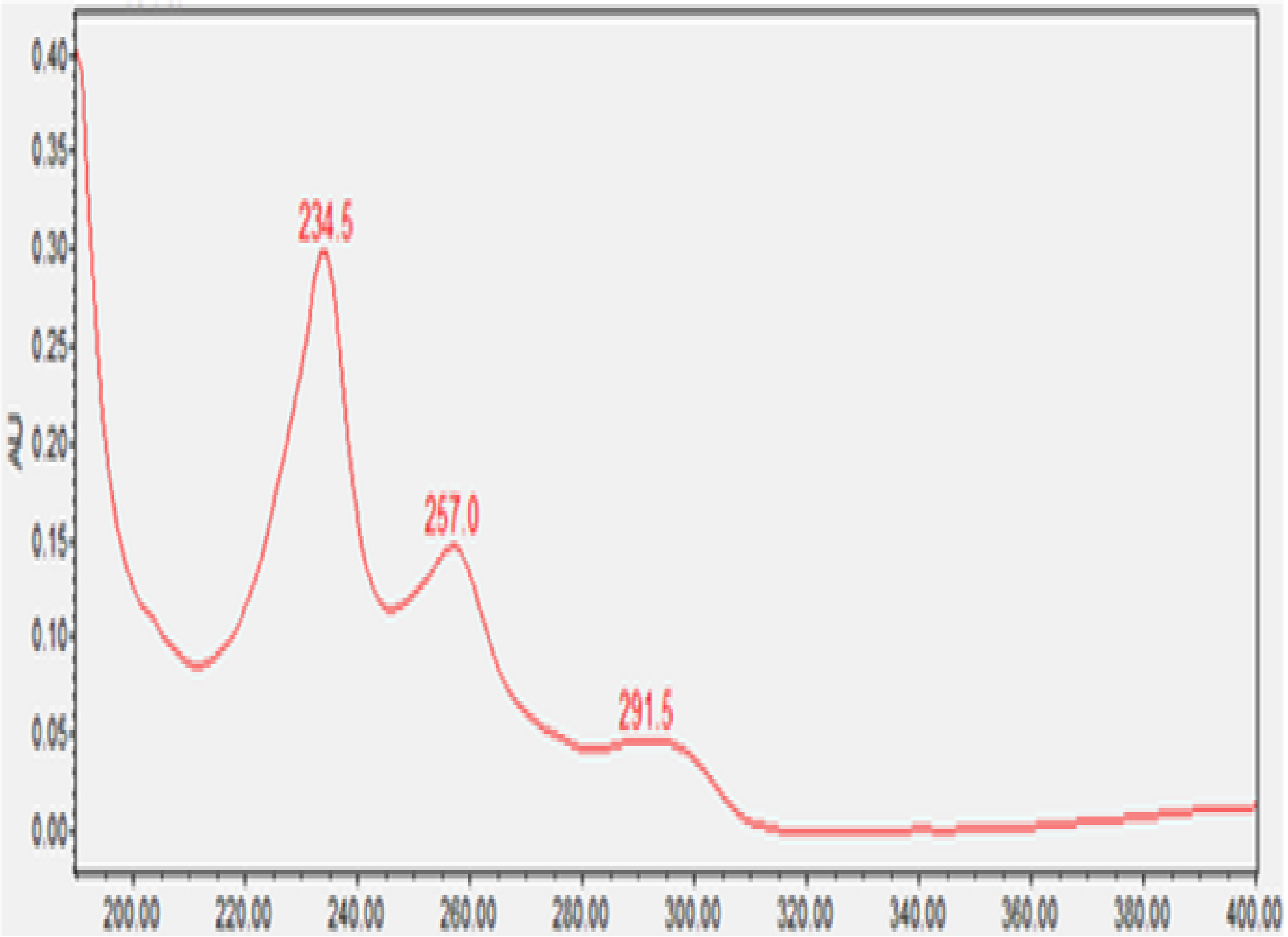

nm

HRESIMS spectrum

Single Mass Analysis
Tolerance = 5.0 mDa / DBE: min = -1.5, max = 50.0
Element prediction: Off
Monoisotopic Mass, Even Electron Ions
1 formula(e) evaluated with 1 results within limits (up to 50 closest results for each mass)
Elements Used:
C: 34-34 H: 41-41 O: 13-13 Na: 0-1

Mass	Calc. Mass	mDa	PPM	DBE	Formula	C	H	O	N...
657.2586	657.2547	3.9	5.9	14.5	C34 H41 O...	34	41	13	

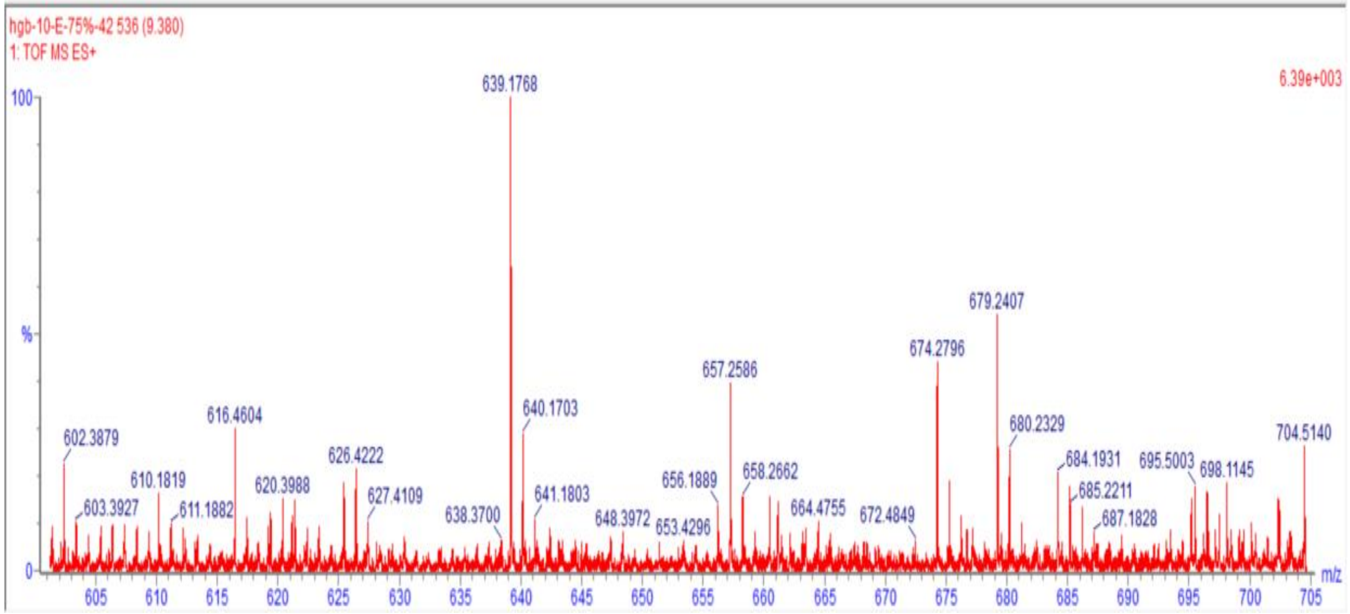

Supplementary 2: Table

Table S1. The ^1H and ^{13}C NMR data of compounds **1-3** in $CDCl_3$ (δ in ppm).

	**1**		**2**		**3**	
**Position**	***δ*C, type**	**δH (*J* in Hz)**	*δ*C, type	δH (*J* in Hz)	*δ*C, type	δH (*J* in Hz)
1	157.9, C		157.7, C		158.6, C	
2	130.1, CH	7.30 d (9.4)	129.5, CH	7.23 m	129.9, CH	7.30 m
3	129.6, CH	7.30 d (9.4)	129.9, CH	7.23 m	130.2, CH	7.30 m
4	158.4, C		158.3, C		158.0, C	
4a	112.8, C		112.8, C		112.5, C	
5	191.1, C		190.9, C		190.8, C	
5a	114.6, C		113.8, C		114.9, C	
6	161.5, C		161.2, C		162.4, C	
6a	137.4, C		134.4, C		131.8, C	
7	142.5, C		142.0, C		142.7, C	
7a	122.0, CH	7.75 s	120.8, CH	7.61 s	120.6, CH	7.72 s
8	131.1, C		130.6, C		132.9, C	
8a	186.3, C		186.3, C		186.0, C	
9	112.7, C		112.7, C		112.7, C	
10	21.4, CH_2	3.03 t (7.5)	20.1, CH_2	3.05 m<br>2.83 m	70.8, CH	5.30 d (2.4)
11	40.5, CH_2	2.80 t (7.5)	28.4, CH_2	2.30 m<br>1.94 m	33.8, CH_2	2.52 m<br>2.37 m
12	210.6, C		71.7, C		71.8, C	
13	36.0, CH_2	2.44 q (7.3)	56.1, CH	3.94 s	57.4, CH	4.13 s
14	8.0, CH_3	1.07 t (7.3)	171.6, C		171.5, C	
15	39.5, CH_2	3.49 s	52.7, CH_3	3.74 s	52.7, CH_3	3.70 s
16	170.8, C		32.5, CH_2	1.61-1.72 m	32.3, CH_2	1.77 m<br>1.51 m
17	52.6, CH_3	3.72 s	6.9, CH_3	1.09 t (7.5)	6.8, CH_3	1.09 t (7.2)
1'					101.5, CH	5.44 d (2.8)
2'					24.6, CH_2	2.02 m<br>1.55 m
3'					24.6, CH_2	2.02 m<br>1.55 m
4'					74.7, CH	3.54 m
5'					67.6, CH	4.11 m
6'					17.3, CH_3	1.23 d (6.9)
1''					99.7, CH	4.84 d (3.4)
